# Annotations

**Published:** 1896-08-01

**Authors:** 


### Annotations.

### A New "Cure" for Drunkenness.

Some little time ago the Hon. Dudley Campbell made himself sponsor for a new cure for what is called the "drink crave." The cure was fruit—vegetarianism, in short. We ventured to point out with some definiteness the necessity for "proof" of this remedial action of fruit. By way of answer, Mr. Dudley Campbell sends us a pamphlet, written by an American medical man, and asks us to accept it as conclusive. But let Mr. Campbell, or any other rational person, think for a moment what this means. It means that we are to consider a case scientifically demonstrated on the authority of one single person, and that person financially interested in getting his theories accepted. We would ask Mr. Campbell, who, we believe, is a barrister, whether the evidence of one single witness, and that witness an interested person, subjected to no cross-examination, would be sufficient to win a verdict in law? If not in law, why in science? The law in mportant cases requires not one witness but several; and not only so, but it employs opposing counsel and solicitors, a judge, or it may be several judges, and a jury; and even when all this has been done, it has sometimes been found impossible to elicit the truth. Now science is at least as difficult as law; and when the conclusions of science have to be converted into life or health conserving rules in medical practice, it is at least as essential that the actual truth should be arrived at as it is in a legal controversy in which no more than two persons may have any direct concern. We must ask Mr. Campbell's permission, in his case of a new cure for the "drink crave," to return at least the Scotch verdict of "Not proven," notwithstanding the pamphlet of the American doctor, in which he seems so profoundly but "unlegally" and unscientifically to believe.

### Obstetric Nurses this Time.

The midwives question seems to be prolific of solutions, and now we have yet another Bill cast upon the world, this time from the prolific pen of Dr. Rentoul. It is not a short one, nor is it very simple, neither of which, perhaps, was to be expected, considering its parentage. One might, however, have expected that at least it would be found to deal with midwives. But not a bit of it. Instead of a Bill to regulate midwives, we find one for the better training of obstetric nurses; and when we inquire what is the nature and character of the strange and unknown beings who go by the name of obstetric nurses, we find that they are not strange at all, and that we are but dealing with our good old friend the monthly nurse! The obstetric nurse is defined as "a person who acts, or is entitled to act, as an obstetric nurse in all cases of labour, under the direct supervision of a registered medical practitioner." But that is not a midwife, the very essence of whose occupation is that she acts by herself, and not under the direct supervision of anyone. Either this curious creature, the obstetric nurse, is a being as yet non-existent, or she is a monthly nurse. But surely there is no over-mastering popular demand for the "supervision" of a class of women who are already under the direct supervision of the medical profession, or for the "registration" of monthly as distinct from any other class of nurses. If Dr. Rentoul and those who see with him are prepared with a scheme for providing every parturient woman with a doctor who shall be sure to arrive in time, let them produce it. If not, let them allow those who wish to improve the education of the midwife to have their say. As a fact, and as part of our social system, the midwife exists here as she does in almost every country in the world, and there is reason to believe that she does great harm, as the result of her operations being entirely uncontrolled. This is the evil that has to be dealt with. The real problem is the control of the midwife; and when people, instead of producing a Bill for that purpose, produce Bills for the registration of midwifery nurses, and obstetric nurses, and such-like curious creatures, they merely show that they do not recognise what is the real question which has to be submitted to the legislature.

### Margarine, Science, and Selfishness.

It used to be thought and taught that the progress of science necessarily meant the progress of man in everything that related to his health, wealth, and happiness. We know better now. It is found by experience that wherever science conflicts with the interests of individual men or classes of men, she must fight as strenuously for her own hand as religion, liberty, or any other antagonist of human selfishness. This little preface is suggested by the experience of the Select Committee appointed to inquire into the working of the Margarine Act, 1887, and the Sale of Food and Drugs Act, 1875, and any Acts amending the same. The committee has found that those Acts are good, and their effects good, conditionally; the condition being that they be duly enforced by the local authorities. But in many cases they are not duly enforced, or not enforced at all by the local authorities; and the reason why they so often fail of being enforced is that the local authorities are so largely leavened by the "local tradesman" element. That is to say, that the most important practical discoveries and advances of science may be rendered absolutely useless by the ignorant selfishness of a few individuals, whose object is to deceive their neighbours by manufacturing and selling something which is different from what it pretends to be. The committee are strongly of opinion "that a local authority which includes even a considerable minority of tradesmen, and which has been found to persistently neglect the enforcement of the Adulteration Acts, should in this matter be superseded by some other authority." This is a serious indictment of the tradesman element in local authorities; and the tradesman here compares very unfavourably with the medical man, who has practically never been known to neglect public sanitation in order to increase general illness and his own emoluments thereby. The committee's recommendation will not fail, we hope, of receiving due attention in the right quarter. It is ridiculous that the country should be at the expense of making costly and scientific investigations into the food question, and of providing machinery to secure the wholesomeness of every variety of food, and that all its efforts should be frustrated by the greedy knavery of a few selfish and wicked persons.

